# Supplementary material for: Propionate Converting Anaerobic Microbial Communities Enriched from Distinct Biogeochemical Zones of Aarhus Bay, Denmark under Sulfidogenic and Methanogenic Conditions
Source: Microorganisms. 2020 Mar 11;8(3):394. doi: 10.3390/microorganisms8030394 (PMC7143418; doi:10.3390/microorganisms8030394)
Supplement: Supplementary file 1 [file microorganisms-08-00394-s001.pdf]

Supplemental Information:

Table 1. Overview of reactions examined in this study. ΔG values were obtained from Thauer et al., 1977.

No.	Equation			ΔG°' (kJ/reaction)*
	**Acetogenic reactions**			
**1**	Propionate$^-$ + 3 H_2O	→	Acetate$^-$ + HCO_3^- + 3 H_2 + H^+	+76.1
	**Sulfate-reducing reactions**			
**2**	Propionate$^-$ + 0.75 SO_4^{2-}	→	Acetate$^-$ + 0.75 HS^- + HCO_3^- + 0.25 H^+	–37.8
**3**	4 H_2 + SO_4^{2-} + H^+	→	HS^- + 4 H_2O	–151.9
**4**	Acetate$^-$ + SO_4^{2-}	→	2 HCO_3^- + HS^-	–47.6
	**Methanogenic reactions**			
**5**	4 H_2 + HCO_3^- + H^+	→	CH_4 + 3 H_2O	–135.6
**6**	Acetate$^-$ + H_2O	→	CH_4 + HCO_3^-	–31.0
	**Syntrophic propionate conversion**			
**1+5**	Propionate$^-$ + 0.75 H_2O	→	Acetate$^-$ + 0.75 CH_4 + 0.25 HCO_3^- + 0.25 H^+	–25.6
	**Complete propionate conversion by SRB**			
**2+4**	Propionate$^-$ + 1.75 SO_4^{2-}	→	1.75 HS^-+ 3 HCO_3^- + 0.25 H^+	–85.4
	**Complete propionate conversion by syntrophs and methanogens**			
**1+5+6**	Propionate$^-$ + 1.75 H_2O	→	1.75 CH_4 + 1.25 HCO_3^- + 0.25 H^+	–56.6

Table S2. Overview of all enrichment slurries fed with propionate and the total amounts of the reactants consumed and products formed during the enrichment period. The enrichment slurries consisted of sediment from either the sulfate zone (SZ), sulfate-methane transition zone (SMTZ) or methane zone (MZ) and were incubated at 25°C or 10°C, with 3 mM, 20 mM or without (-) sulfate amendments along the study. The slurries P1/P2, P3/P4, P5/P6, P7/P8 from each sediment zone are biological replicates. Slurries with * are presented in the propionate conversion graphs and used for molecular analysis.

Origin	Slurry code	Treatment	Incubation temperature (°C)	Reactants (µmol/slurry)		Products (µmol/slurry)		
				Propionate	Sulfate	Acetate	Sulfide	Methane
SZ	*SZP1	-	25	26157	336	17905	881	37464
	SZP2	-	25	2726	159	1183	284	0
	SZP3	20 mM SO_4^{2-}	25	20623	29592	19126	29062	33
	*SZP4	20 mM SO_4^{2-}	25	26681	42456	25677	39089	1038
	SZP5	-	10	17190	354	13170	151	9083
	*SZP6	-	10	18414	125	9558	207	14959
	SZP7	20 mM SO_4^{2-}	10	15491	13410	18442	18296	0
	*SZP8	20 mM SO_4^{2-}	10	17679	18592	21716	21495	0
SMTZ	SMTZP1	3 mM SO_4^{2-}	25	27494	9343	25160	6993	29829
	*SMTZP2	3 mM SO_4^{2-}	25	32207	9330	34093	8892	15509
	*SMTZP3	20 mM SO_4^{2-}	25	27819	37381	9246	34439	339
	SMTZP4	20 mM SO_4^{2-}	25	28694	40125	11250	35587	74
	*SMTZP5	3 mM SO_4^{2-}	10	18465	6638	16996	7316	2797
	SMTZP6	3 mM SO_4^{2-}	10	17365	6839	17427	7642	2197
	*SMTZP7	20 mM SO_4^{2-}	10	26008	33660	27709	34299	17
	SMTZP8	20 mM SO_4^{2-}	10	27162	35346	26523	36833	34
MZ	*MZP1	-	10	7204	932	7346	552	3909
	MZP2	-	10	3897	809	1575	673	926
	*MZP3	-	25	27472	825	13791	563	38473
	MZP4	-	25	34576	604	20771	509	43969
	MZP5	20 mM SO_4^{2-}	25	39414	33244	30817	32821	1670
	*MZP6	20 mM SO_4^{2-}	25	49211	45738	49783	40572	418
	MZP7	20 mM SO_4^{2-}	10	16577	20444	15542	15184	0
	*MZP8	20 mM SO_4^{2-}	10	11366	16233	11913	12356	0

Table S3. The number of reads per sample generated by Pyrosequencing for Bacteria and HiSeq Illumina sequencing for Archaea. ENV: Environmental sample.

Origin	Slurry code	Bacterial reads	Archaeal reads
Sulfate zone	ENV	8733	9120
	SZP1	9903	13481
	SZP4	12305	74345
	SZP6	27196	67941
	SZP8	15062	16472
Sulfate-methane transition zone	ENV	3186	18696
	SMTZP2	8730	33641
	SMTZP3	5653	30858
	SMTZP5	5949	79958
	SMTZP7	3934	21631
Methane zone	ENV	10824	30848
	MZP1	14192	78094
	MZP3	14752	108810
	MZP6	14171	25156
	MZP8	1888	5547

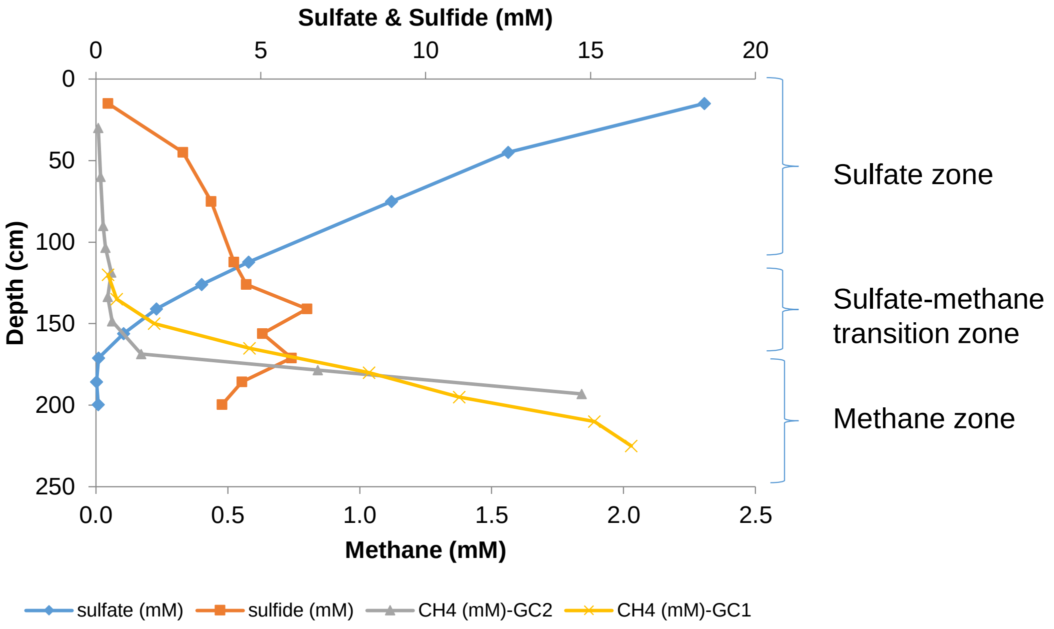

Figure S1. Depth profiles of sediment pore water sulfate, sulfide and methane for Station M1, in Aarhus Bay, Denmark. Methane-GC1 and Methane-GC2 stands for methane concentrations retrieved from two different gravity corers, gravity corer 1 and 2, respectively. SZ; Sulfate zone, SMTZ; sulfate-methane transition zone; MZ, methane zone.

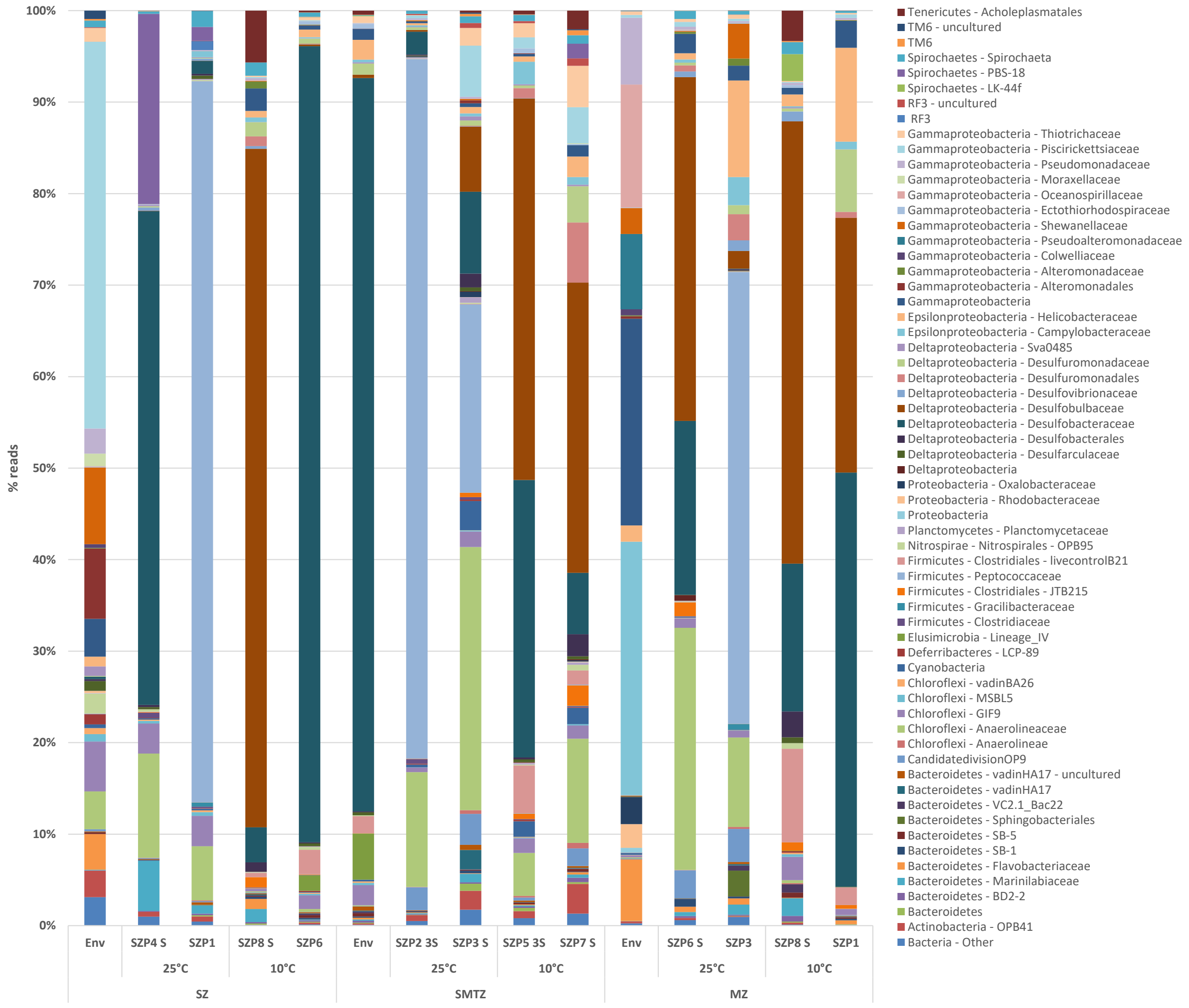

Figure S2. Relative abundance of the bacterial community in all slurries and environmental samples at family level, normalized to 100%. Only those families that were present at an abundance >1% in at least one sample were included in the graph. SZ: Sulfate zone, SMTZ: Sulfate-methane transition zone; MZ: Methane zone. Env: Sediment sample belonging to the indicated biogeochemical zone. S: 20mM sulfate, 3S: 3mM sulfate is used as electron acceptor in slurries. Slurries that were not labeled with 'S' or '3S' were incubated without sulfate.

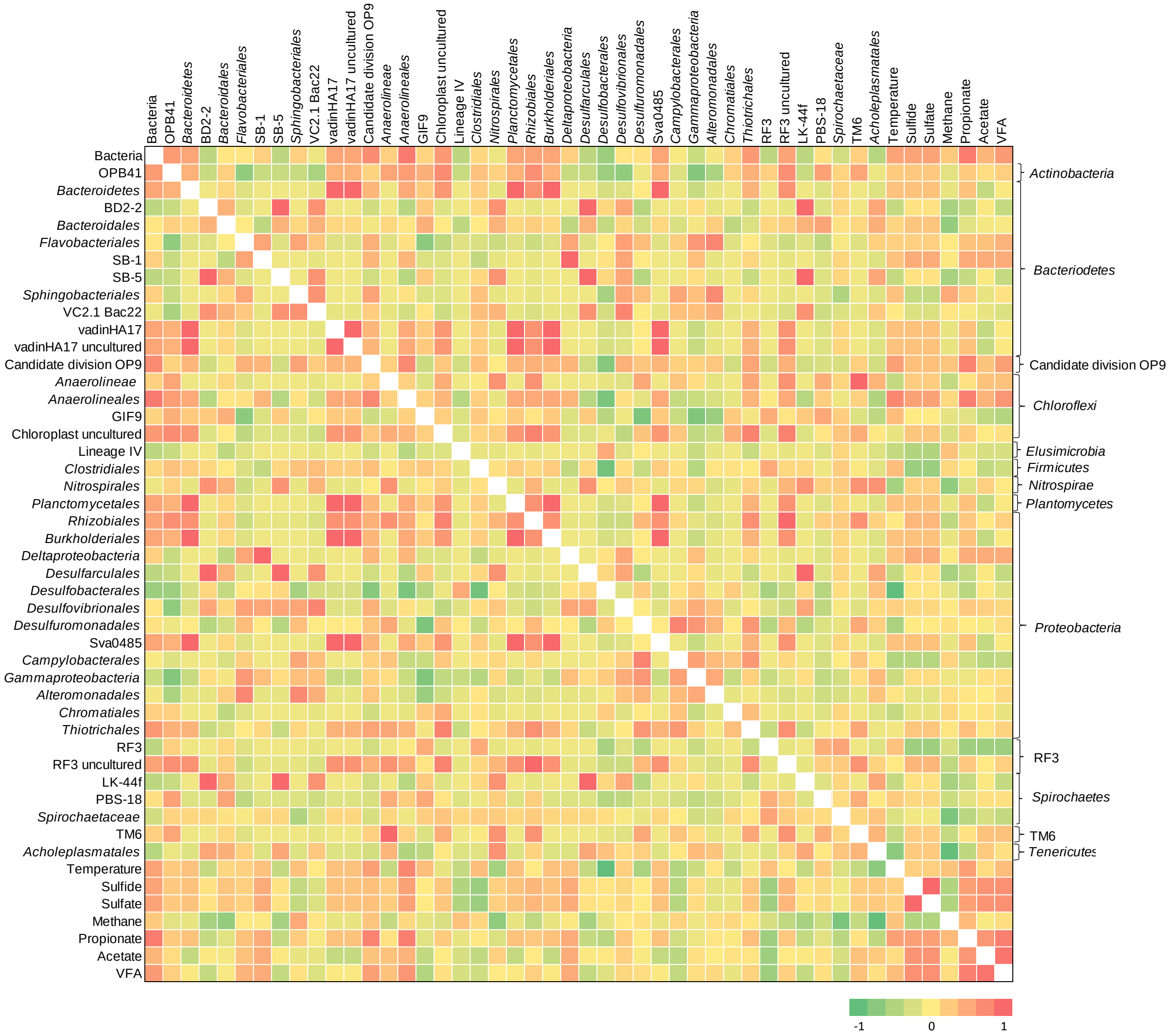

Figure S3. Heatmap depicting the correlation between bacterial orders present at a relative abundance >1% of total reads across the 12 slurry samples analyzed and experimental parameters. Correlations were determined by means of the two tailed Spearman's Rank Order Correlation test. The heatmap colors shifted towards red indicate strong correlation.

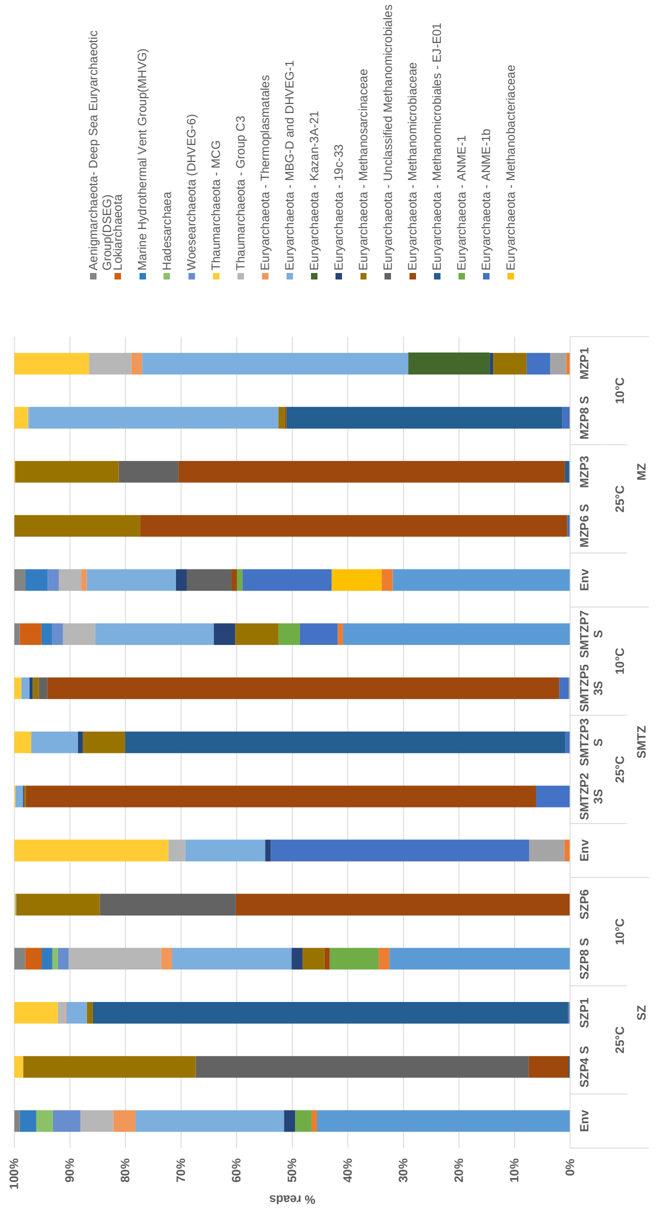

Figure S4. Relative abundances of the archaeal community in all slurries and environmental samples at family level, normalized to 100%. Only those families that were present at an abundance >1% in at least one sample were included in the graph. SZ: Sulfate zone, SMTZ: Sulfate-methane transition zone; MZ: Methane zone. Env: Sediment sample belonging to the indicated biogeochemical zone. S: 20mM sulfate, 3S: 3mM sulfate is used as electron acceptor in slurries. Slurries that were not labeled with 'S' or '3S' were incubated without sulfate.

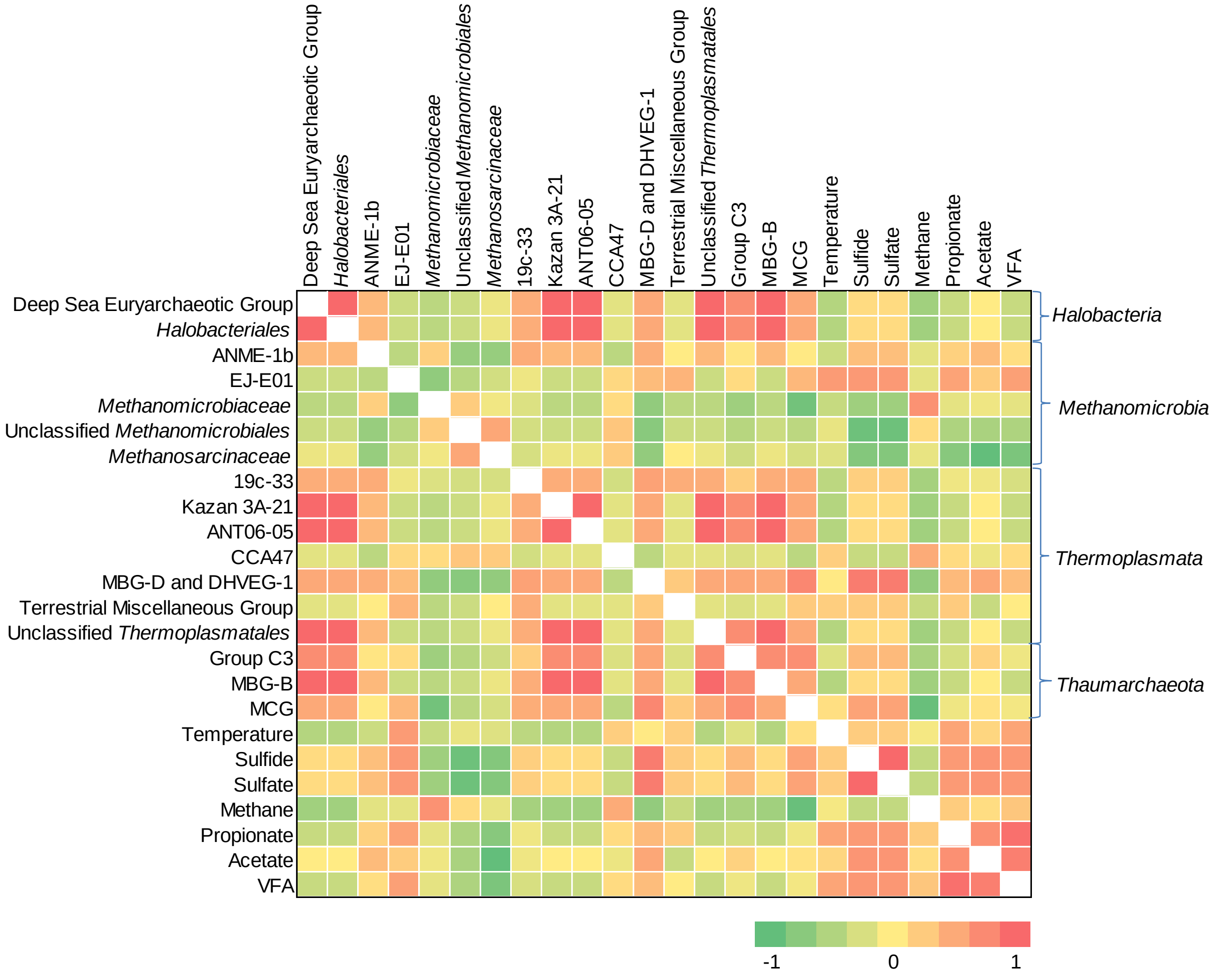

Figure S5. Heatmap depicting the correlation between archaeal families present at a relative abundance >1% of total reads across the 12 slurry samples analyzed and experimental parameters. Correlations were determined by means of the two tailed Spearman's Rank Order Correlation test. The heatmap colors shifted towards red indicate strong correlation.
